# Comparative analysis of combined spinal–epidural anesthesia and general anesthesia in percutaneous nephrolithotomy: a prospective study on surgical team and operating room personnel satisfaction

**DOI:** 10.1007/s00345-024-04977-4

**Published:** 2024-04-26

**Authors:** Salih Bürlukkara, Afife Ayla Kabalak, Alpay Ateş, Özer Baran, Aykut Aykaç, Hakkı Uğur Özok

**Affiliations:** 1 Department of Urology, Karabuk University Medikal Faculty, Karabuk, Turkey; 2 Department of Anesthesiology, Karabuk University Medikal Faculty, Karabuk, Turkey; 3 Department of Urology, Eskişehir City Hospital, Eskişehir, Turkey

**Keywords:** Kidney, Stone, Percutaneous, Anesthesia

## Abstract

**Objective:**

We aimed to investigate the efficacy and complications of combined spinalepidural anesthesia and general anesthesia in percutaneous stone surgery prospectively.

**Materials and methods:**

The study prospectively included patients who underwent percutaneous nephrolithotomy with general anesthesia (Group.1) or combined spinal–epidural anesthesia (Group.2) at the Department of Urology, Training and Research Hospital, Karabuk University. between December 2018 and December 2019. The effect of the anesthesia technique on the comfort and satisfaction of the operating room personnel, surgeon and anesthesia team were prospectively evaluated and recorded.

**Results:**

During the postoperative period, the spinal anesthesia group had a significantly lower visual analog score than the general anesthesia group. No patient in either group required narcotic analgesics during the postoperative period. In terms of overall satisfaction scores, the surgeon performing the surgical procedure had a significantly higher satisfaction score in the general anesthesia group than in the CSEA group. The score was considered good in the general anesthesia group and moderate in the CSEA group. Personnel satisfaction was higher in the patient group that underwent CSEA. In the general anesthesia group, the score was considered to be average. In the CSEA group, the satisfaction score was considered good, with a statistically significant difference (*p* < 0.05). The anesthesia team’s satisfaction score was moderate, with no significant difference between the CSEA and general anesthesia groups (*p* > 0.05).

**Conclusion:**

PCNL under CSEA can be performed safely in certain individuals. Different anesthetic techniques may have varied levels of satisfaction among the surgical team.

## Introduction

According to the European Urological Association guidelines, percutaneous nephrolithotomy (PCNL) is the recommended primary treatment for stones larger than 20 mm. PCNL is beneficial in patients with multiple and intrarenally positioned stones because it allows for successful stone removal with minimal kidney damage. In the prone, supine, or modified supine position, the surgery can be performed using general anesthesia or combined spinal–epidural anesthesia (CSEA).

Percutaneous nephrolithotomy is helpful because it enables successful stone removal. Several changes and modifications have been made during the last 2 decades to reduce morbidity, analgesic requirements, and hospital stay duration [1]. According to the literature, general anesthesia has an advantage over regional anesthesia in terms of hemodynamic control, airway control, and patient and surgeon satisfaction. This advantage is more pronounced in patients who require upper pole puncture [2, 3]. On the other hand, CSEA reduces the risk of complications using less invasive surgical methods [4]. Percutaneous surgery is a minimally invasive surgical intervention that, when combined with regional techniques, is thought to improve patient safety, early recovery, and discharge [4–6]. The English literature has no studies monitoring and evaluating the satisfaction of the surgical team, operating room personnel, and anesthesia team in PCNL performed under CSEA and general anesthesia. Our study is the first in this field.

In this prospective study, we aimed to investigate the effect of combined spinal–epidural anesthesia and general anesthesia on the comfort and satisfaction of operating room personnel, surgeons, and anesthesia team during percutaneous stone surgery in terms of effectiveness and complications. We aimed to investigate the satisfaction scores of anesthesiologists, surgeons, and operating room personnel, duration of anesthesia and surgery, intravenous and epidural analgesic requirements, patient satisfaction at 24 h postoperatively, postoperative analgesia requirements, mobilization times, and the effect of both methods on surgical success and determine the appropriate anesthesia technique in patients undergoing general anesthesia and CSEA.

## Materials and methods

The study prospectively included patients who underwent percutaneous nephrolithotomy with general anesthesia (group 1) or combined spinal–epidural anesthesia (group 2) at the Department of Urology, Training and Research Hospital, Karabuk University, between December 2018 and December 2019, as well as operating room personnel, surgical team, and anesthesia team.

This study was approved by the Institutional Review Board and registered with Clinical Trials. All participants provided their informed consent to participate in the study. Our primary outcome was to ensure that the surgeon, anesthesiologist, and operating room personnel were satisfied with the patients undergoing CSEA and general anesthesia. Secondary outcomes included postoperative pain evaluated using the visual analog scale (VAS). A two-tailed error of 5% and a *β* error of 10% were acceptable for identifying a 1.5-point difference in the VAS pain scale between the CSEA and general anesthesia groups. According to these estimates, the required sample size for each arm was 34 patients, and the study was stopped after 44 patients were enrolled.

The surgical procedure was performed by five experienced urologists, and anesthesia was provided by two anesthesiologists. Satisfaction of the operating room personnel was obtained from two different personnel. The surgical team performing the surgical procedure was asked to score their satisfaction with the ease of patient positioning, placement of the pelvic floor system, and overall comfort immediately after surgery on a numerical scale (0–10). Similarly, the anesthesia team and operating room personnel were asked to indicate their satisfaction with the surgery on a scale of 1 (lowest) and 10 (highest). All data were collected and kept at the patient’s bedside. Scores of 0 to 2 were classified as very poor, 3–5 as poor, 6–8 as moderate, and 9–10 as good.

Patients who had uncontrolled diabetes mellitus, hypertension, cardiac arrhythmia, obesity, severe pulmonary disease, liver failure, coagulation disorder, cerebrovascular disease, and refusal of combined spinal–epidural anesthesia were excluded. The patients were divided into two groups: conventional general anesthesia and epidural anesthesia.

Patients who agreed to participate in the study were transferred to the preoperative area approximately 1 h before surgery. Our hospital had a separate anesthesia team that performed CSEA, and group assignment was made by the team performing CSEA. Depending on the patient’s current medical history and condition, this team determined and divided the anesthetic procedure into groups.

All patients’ demographic data, stone localization size and density, urinary anomalies (horseshoe kidney and pelvic kidney), intraoperative data (procedure time, scope time, number and localization of access, anesthetic requirement and dose, and blood transfusion), postoperative findings (length of hospital stay, mobilization, analgesia requirement, blood transfusion, complications, and residual stone rate), and medical records, including preoperative, perioperative, and postoperative data were prospectively evaluated and recorded.

The VAS of each patient was evaluated and recorded 6 h after the effect of CSEA had gone in the postoperative period.

### General anesthesia technique

All patients administered general anesthesia had intravenous (iv) vascular access, and baseline values for heart rate, blood pressure, and oxygen saturation were collected during regular monitoring. After 3 min of 100% oxygen via mask preoxygenation, 2- to 3-mg/kg propofol, 1-mcg/kg fentanyl, and 0.6-mg/kg rocuronium were administered intravenously to induce anesthesia. Anesthesia was maintained with 5–6% desflurane and a mixture of 50% O2 and 50% N_2_O, and mechanical ventilator settings were adjusted. At the end of the surgical procedure, the maintenance medications were completely turned off, and 100% O2 ventilation was initiated. When spontaneous breathing began, the reverse was performed, and the patient was extubated after adequate spontaneous breathing was achieved.

### Combined spinal–epidural anesthesia technique

In the preoperative preparation room, all patients undergoing spinal anesthesia were premedicated with 0.03-mg/kg *iv* midazolam premedication by intravenous infusion of Ringer’s lactate solution for preoperative hydration. At the end of this period, the basal values of patients who were taken to the operating room and routinely monitored were recorded. After appropriate sterilization in the sitting position, a 25-G, 90-mm pencil point spinal atraumatic insertion in the lumbar region at the L3–L4 or L4–L5 level was performed, followed by 10- to 15-mg 0.5% bupivacaine, and free cerebrospinal fluid flow was observed.

### Surgical technique

In both groups, the ureteral orifice on the same side was visualized, and a 6-Fr ureteral catheter was inserted into the ureter following ureteroscopy and fixed to the urethral catheter. The patients were then placed in the prone position. After administering diluted radio-opaque material from the ureteral catheter, an 18-gauge percutaneous access needle was used under C-arm fluoroscopy to gain access to the designated calyx, and a 0.038-in. guidewire was advanced into the renal collecting system. The guidewire was used to widen the access site to a 30-Fr Amplatz dilator, after which a sheath (30 Fr) was placed. The renal collecting system was accessed with a 26-Fr nephroscope, and the stones were fragmented with a pneumatic lithotripter and extracted using a grasper. Stone-free status was confirmed by direct examination or fluoroscopy. Additional access was performed if single access was inadequate to achieve stone-free status in patients with stones in different calyces. After the operation, a 14-Fr nephrostomy tube was inserted through the access sheath, and the procedure was terminated when the alexia system was examined using fluoroscopy. Stones of 4 mm on the postoperative direct cystogram were considered clinically insignificant. Complications were graded using the Clavien classification.

### Statistical analysis

The entire study was conducted using the SPSS 17.0 statistical package program. The conformity of numerical variables to normal distribution was determined using the Kolmogorov–Smirnov test. Categorical variables were expressed as frequency and percentage, and numerical variables were expressed as mean, standard deviation, median, and minimum–maximum values. Two independent means were compared using the Student’s *t* test, and two independent medians were compared using the Mann–Whitney *U* test. The relationship between two independent categorical variables was investigated using the chi-squared (Fisher’s exact) test. The study was conducted at a 95% confidence level (*p* < 0.05 indicated a statistically significant difference).

## Results

The study included 126 patients: 79 (68.1%) males and 37 (31.9%) females. The general anesthesia group (group 1) had 72 (62.1%) patients, and the combined spinal–epidural anesthesia group (group 2) had 44 (37.9%) patients. A comparison of the operative data for both groups is shown in Table 1.

**Table 1:** Demographic data, intraoperative and pre-postoperative parameters of the patients and comparison of overall satisfaction score

	Group 1 (GA)	Group 2 (CSEA)	*p*
Male/female (number)	48 (60.8%)/24 (64.9%)	31 (39.2%)/13 (35.1%)	0.671
Age (mean ±)	50.69 ± 13.9	53.18 ± 11.21	0.319
BMI	28.25 ± 4.61	29.04 ± 5.7	0.276
Stone load (mm)	30.15 ± 10.6	31 ± 13.7	
Stone size (mm)	2.28 ± 1.64	2.16 ± 1.51	0.912
Density (HU)	1051.46 ± 332.3	1046.68 ± 398.17	0.946
Stone direction right/left (number, %)	32 (65.3%)/40 (59.7%)	17 (34.17%)/27 (40.13)	0.539
Double J/re-entry	10 (13.9%)/62 (86.1%)	6 (13.6%)/38 (86.4)	0.969
Fluoroscopy time (sec)	4.15 ± 1.61	3.93 ± 1.56	0.603
Operation time (min.)	123.9 ± 50.3	130.9 ± 41.9	0.298
Hospitalization duration(days)	4.25 ± 1.98	4.29 ± 1.48	0.493
Stone-free rate (number, %)	47 (65.3%)	30 (68.2%)	0.748
Satisfaction score			
Surgeon score	9	7	< 0,05
Personnel score	6	9	< 0,05
Anesthesia score	8	8	> 0,05

The mean age of the patients in the general anesthesia group was 50.69 year, and the mean age of the patients in the group receiving combined spinal–epidural anesthesia was 53.18 year, with both groups having comparable mean ages. There were 48 male and 24 female patients in group 1 and 31 male and 13 female patients in group 2. The body mass index (BMI) was 28.25 in group 1 and 29.04 in group 2, with no significant difference between the two groups.

There was no statistically significant difference between the two groups in terms of mean stone size (mm), number of stones, stone direction (right/left), and operation time (min; (*p* > 0.05). Group 1 had a mean operation time of 123.9 ± 50.3 and a fluoroscopy time of 4.15 ± 1.61, whereas group 2 had a mean operation time of 130.9 ± 41.9 min and a fluoroscopy time of 3.93 ± 1.56, with no statistically significant difference. Patients with stones smaller than 4 mm on the early postoperative renal ureter bladder radiograph and nonopaque whole abdominal computed tomography taken at the control examination 1 month later were considered stone-free; the rate was 65% in group 1 and 68% in group 2. Stone-free rates were comparable in both groups, with no statistically significant difference between the two groups.

The patients had a preoperative stone density of 1,051.46 HU in group 1 and 1,046.68 HU in group 2. There was no statistically significant difference between the two groups in terms of stone density (*p* = 0.946).

There was no significant difference between the two groups in terms of access number and location. Because four patients in group 1 and three patients in group 2 had preoperative nephrostomy catheters, access was performed via the catheter. Only one patient in group 2 had intercostal access.

The postoperative hospital stay duration was 4.25 days in group 1 and 4.29 days in group 2, with no significant difference between the two groups. After consultation with interventional radiologists, one patient from each group was referred to an external center for embolization due to uncontrollable bleeding.

While ten patients in group 1 underwent double J stenting postoperatively, only six patients in group 2 did, and the difference was not statistically significant. High residual stone load and prolonged drainage were the primary reasons for postoperative double J stenting.

During the postoperative period, the spinal anesthesia group had a significantly lower visual analog score than the general anesthesia group. No patient in either group required narcotic analgesics during the postoperative period.

In terms of overall satisfaction scores, the surgeon performing the surgical procedure had a significantly higher satisfaction score in the general anesthesia group than in the CSEA group (Table 1). The score was considered good in the general anesthesia group and moderate in the CSEA group (*p* < 0.05).

Personnel satisfaction was higher in the patient group that underwent CSEA. In the general anesthesia group, the score was considered to be average. In the CSEA group, the satisfaction score was considered good, with a statistically significant difference (*p* < 0.05). The anesthesia team’s satisfaction score was moderate, with no significant difference between the CSEA and general anesthesia groups (*p* > 0.05).

Complications in the groups were determined using the modified Clavien classification system. According to Clavien’s score, no grade 3b, 4, or 5 complications were observed in either group. There was no significant difference in mean hemoglobin drop between the two groups. Blood transfusions were performed in four (5.6%) patients in group 1 and three (6.8%) patients in group 2. One patient in each group was scheduled for embolization due to uncontrolled bleeding, and planned referrals were made after consultation with interventional radiologists. There were no major vessel or visceral organ injuries in either group. On the first postoperative day, one patient in group 1 had atelectasis because of fever and was taken to respiratory rehabilitation.

## Discussion

Although general anesthesia is the most often used method for PCNL in many centers, it may not be the best option for patients with cardiovascular or pulmonary problems. General anesthesia is a challenging method due to the possibility of fluid absorption, dilutional anemia, hypothermia, or significant blood loss [5, 7, 8]. Additionally, general anesthesia carries inherent risks, such as increased incidence of anaphylaxis due to multiple medications, atelectasis, nausea, vomiting, vascular and neurological complications, and endotracheal tube-related problems during the transition from lithotomy to the prone position [5, 9, 10]. Recent studies have shown that CSEA is a viable alternative to general anesthesia, with a favorable hemodynamic profile and lower cost [11]. CSEA is a safe, feasible, and well-tolerated treatment option, particularly for elderly patients with cardiac or pulmonary comorbidities [12]. CSEA is a viable option for PCNL, with favorable and tolerable results, particularly in patients at high risk of general anesthesia and difficult intubation [5].

In a study by Kuzgunbay et al. [4] comparing PCNL surgeries performed under spinal and general anesthesia, there was no significant difference in surgical parameters, such as operation time, fluoroscopy time, hemoglobin drop, and hospital stay length. There was also no difference in postoperative stone-free rates between the two groups.

According to the study of Movasseghi et al. [13], epidural anesthesia is a faster and safer method of anesthesia in PCNL surgeries. Epidural anesthesia improves hemodynamic and hemostatic stability, fewer complications, lower analgesic requirement, and shorter operation time.

In a meta-analysis of 1954 patients by Chunxiao et al., epidural anesthesia and general anesthesia data were analyzed. The two groups showed no significant difference in stone-free rate and PCNL-related postoperative complications. PCNL under CSEA has advantages over general anesthesia in terms of surgical time, hospital stay duration, fluoroscopy time, blood transfusion, postoperative pain, and analgesic requirement. It was determined that CSEA may be a better option for general anesthesia due to improved hemodynamic control and lower cost [11]. Similar to previous studies, we found no significant difference in stone-free rates, length of hospital stay, hemoglobin drop, and transfusion requirement between the combined spinal–epidural and general groups.

Longer anesthesia preparation, intubation, and postoperative recovery time may have resulted in longer operation times in the general anesthesia group. Additionally, in the combined spinal–epidural anesthesia group, the prone position was administered shorter because communication with the patient during the positioning phase may have also reduced operation time. The most critical factors determining surgical success and stone-free rates are renal anatomy, the size and location of the stone, and the surgeon’s experience.

In another randomized controlled study of 858 patients, Xiaocheng et al. [14] found no significant difference between the groups regarding stone-free, blood transfusion, and postoperative fever rates. They also showed that PCNL under epidural anesthesia may reduce operation time, hospital stay duration, and analgesic requirement.

In conclusion, patients administered with CSEA had a shorter operation time, a lower postoperative visual analgesic score, a lower analgesic requirement, a shorter hospital stay, fewer blood transfusions, and lower complication rates than those administered with general anesthesia [2, 15].

The findings of our study showed that the postoperative surgical success and complication results of PCNL surgeries performed with CSEA and GA are comparable. According to the results of our study, spinal anesthesia is a fast and safe method of anesthesia in selected patients for PCNL surgery.

It was found that the satisfaction score of the surgical team was good and higher in the group receiving general anesthesia, and the satisfaction score of the operating room personnel was good and higher in the group receiving combined spinal anesthesia.

The surgeon satisfaction score was higher in the general anesthesia group because of the continuation of the patient’s respiratory movement, decreased block level, increased patient mobility, and pain sensation with prolonged operation time in the CSEA patient group.

The higher personnel satisfaction in the CSEA patient group was attributed to the patient’s cooperation and support with active movements.

In the CSEA group, the measured low VAS score was evaluated as the continuing effect of regional anesthesia. However, no patient required additional analgesics.

The disadvantages of this technique in terms of anesthesia were that a very high-level block was required to relieve renal pain in CSEA, distension of the renal pelvis during PCNL could cause a vasovagal reaction that could not be prevented by regional anesthesia, and increasing the level of the block could lead to respiratory and circulatory system problems during the operation and patient turning.

Disadvantages of PCNL performed under general anesthesia included displacement of the intubation tube during patient positioning, dislocation of the vascular access, disconnection of the patient monitoring cables, and failure to recognize prone-specific eye, neck, and brachial nerve complications. Therefore, the anesthetic score was similar across the two techniques.

In this regard, our study is significant because it is the first to assess surgeon, anesthesiologist, and personnel satisfaction, as well as a pioneer for novel studies and surgical techniques.

### Limitations

This study has several notable limitations. VAS scores are susceptible to individual and subjective interpretation, complicating pain assessment. VAS score may change because the effect of CSEA varies from patient to patient and the effects of regional anesthesia are variable. Pain and patient movement may complicate the surgical procedure in the patient group undergoing CSEA. As the operation time in CSEA increases, the procedure may be unable to continue because of pain, and the surgery may be discontinued. Some patients with Foley and nephrostomy catheters may have reported inflated VAS scores for nonsurgical site pain.

Furthermore, although many studies compare CSEA with general anesthesia, none has compared the two groups regarding satisfaction. This was considered the strength of our study.

## Conclusion

In conclusion, PCNL under CSEA can be performed safely in certain individuals. Different anesthetic techniques may have varied levels of satisfaction among the surgical team. While general anesthesia improves surgeon satisfaction, CSEA increases operating room personnel satisfaction. Further research with a larger series is needed. We anticipate that our study will serve as a foundation for future research.

## Data Availability

The raw data for the dataset is not publicly available to protect the privacy of individuals under the European General Data Protection Regulation. The data is stored on the hard disc and will be shared with you if you wish.
